# Giant Epidermoid Cyst of the Posterior Fossa

**DOI:** 10.7759/cureus.20923

**Published:** 2022-01-04

**Authors:** Rishika Trivedi, Pankaj Trivedi, Rekha Gupta

**Affiliations:** 1 Medicine, Himalayan Institute of Medical Sciences, Dehradun, IND; 2 Neurosurgery, Vasal Hospital Neuro and Trauma Centre, Jalandhar, IND; 3 Pathology, Government Medical College, Amritsar, IND

**Keywords:** ectodermal inclusion cysts, benign cystic lesions, suboccipital craniotomy, posterior fossa, giant epidermoid cyst, cerebellopontine angle epidermoid

## Abstract

Epidermoids are rare intracranial neoplasms that grow slowly and present in the third to fifth decade of life. Giant epidermoid cysts are infrequent, and their occurrence in the posterior fossa is rare. We describe a similar case, where a patient presented with a long-standing history of headache, imbalance, and progressive weakness in the arms. Imaging revealed a giant space-occupying lesion in the posterior fossa measuring 6.25 cm x 7.56 cm x 6.8 cm, which was confirmed on histopathology to be an epidermoid cyst. The patient underwent suboccipital craniotomy extending up to the rectosigmoid junction to remove the same and was on a follow-up to check for recurrences.

## Introduction

Epidermoids are slow-growing and benign cystic lesions. These are rare intracranial congenital neoplasms that account for 1% of all brain tumors [1,2]. Even though the cerebellopontine (CP) angle is the most common location for its occurrence, it accounts for only 7% of the tumors in this region [3]. These are considered to arise from the migration of ectodermal elements during embryogenesis which results in cutaneous epithelium being trapped within neural tissue resulting in the formation of ectodermal inclusion cysts [4]. Only a few lesions reach or exceed the size of 5 cm and are referred to as giant epidermoid cysts (GECs). GECs are discovered in the third to fifth decade of life as the growth rate of this tumor is slow. Hence, the symptoms of compression or irritation of vascular or neurological structures become apparent later [5]. To the best of our knowledge, there have been 26 reported cases that met the criteria of GECs, and only 12 cases of GECs have been reported in the posterior fossa [6,7]. We present a rare case of GEC measuring 6.25 cm x 7.56 cm x 6.8 cm.

## Case presentation

**Figure 1:**
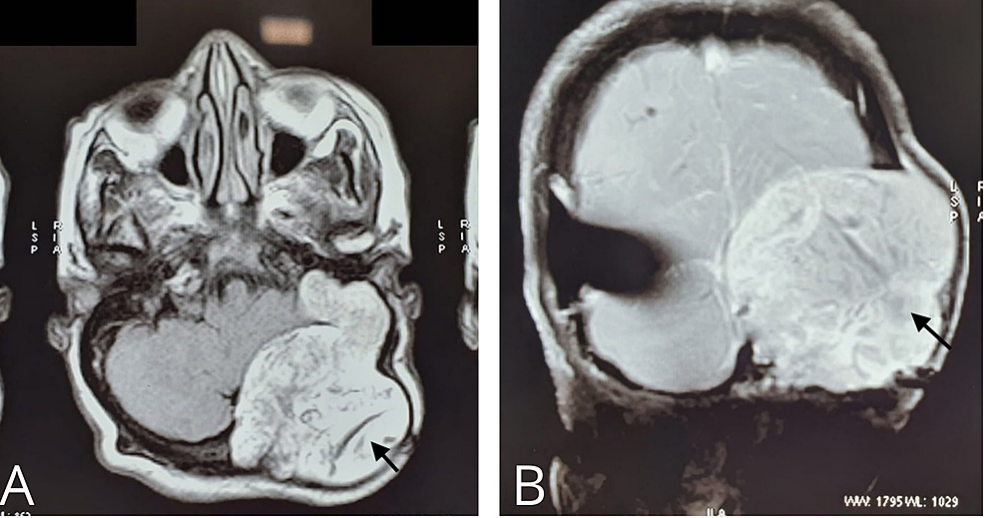
T1-weighted axial (A) and coronal (B) MRI demonstrating a space-occupying lesion with mixed-signal intensities.

A 65-year-old female presented with the chief complaints of headache, which occurred intermittently for 1.5 years, difficulty in walking leading to imbalance since last six months, and gradually progressive weakness in both arms since last four months. MRI of the brain with contrast revealed a large smooth extra-axial lesion in the left posterior occipital convexity measuring 6.25 cm x 7.56 cm x 6.8 cm. The lesion was also extending into the jugular foramen, left CP angle reaching up to the floor of the middle ear (Figure 1). 

**Figure 2:**
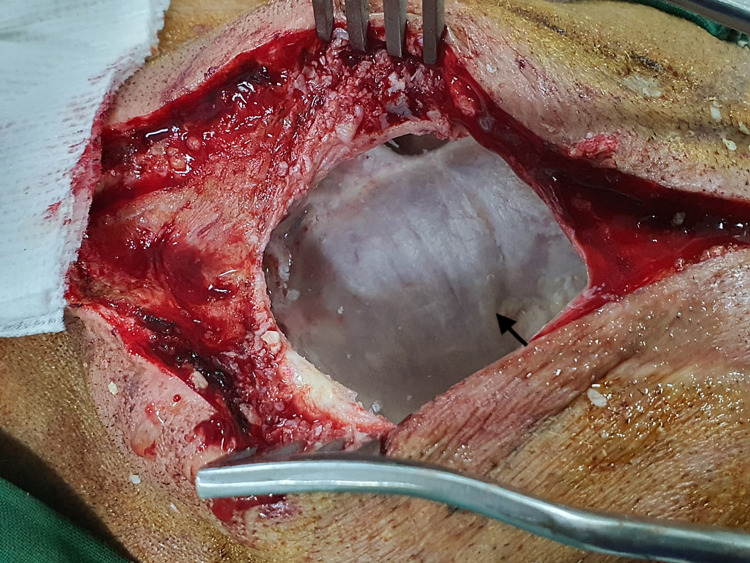
Intra-operative view showing thinned-out dura after removal of tumor (epidermoid).

The patient underwent suboccipital craniotomy extending up to the rectosigmoid junction. A postauricular, paramedian incision measuring 8-10 cm was made for the same. The bone was affected and badly thinned out, hence, cranioplasty was planned for later on as the cerebellum was compressed and time was given for it to expand to occupy its normal place. Finally, a near-total excision of the tumor was performed, except at the CP angle where the capsule was badly adherent to the dura (Figure 2). Gross examination revealed a rounded and slightly fluctuant mass with a smooth whitish capsule. There was no postoperative deficit or cerebrospinal fluid (CSF) leak. 

**Figure 3:**
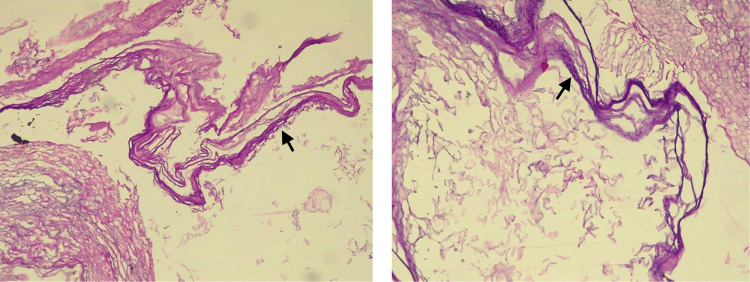
Histology revealed cyst lined by keratinized stratified squamous epithelium and surrounded by a layer of irregular keratin material indicating an epidermoid cyst

**Figure 4:**
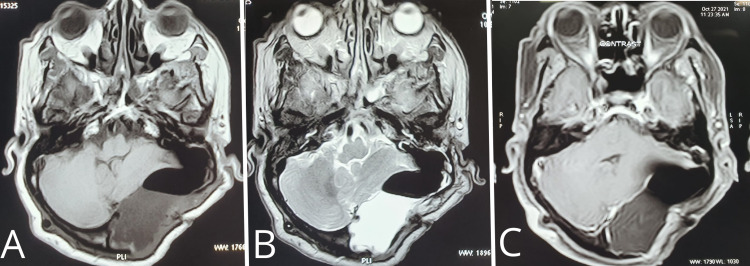
Postoperative MRI of T1-weighted axial section (A), T2-weighted axial section (B), T1-weighted contrast-axial section (C).

The histopathological report showed sheets of keratin with dilated cystic space lined by stratified squamous epithelium. There was no atypia or brain parenchyma identified (Figure 3). The patient was followed monthly for six months and then yearly for two years and showed no signs and symptoms of recurrence of the tumor on clinical examination and imaging (Figure 4). The patient is living an independent life.

## Discussion

Giant epidermoid cysts are rare and benign tumors that can occur at any location in the brain. The most common locations of their occurrence include cerebellopontine angle and temporal and suprasellar regions [8]. Symptomatology depends upon the location of the tumor. Most commonly, cranial nerve palsies of V, VII, VIII, and IX are observed, followed by headaches and seizures. These symptoms are due to irritation of the nerves or pressure on the brain parenchyma [7]. Epidermoid cysts are congenital lesions that grow over time hence remain clinically silent for the majority of the patients, and the symptoms become apparent in adulthood, usually in the third to fifth decade of life [5,9]. Microscopically, epidermoid cysts possess a capsule that consists of a layer of stratified squamous epithelium tissue which appears white and pearly macroscopically. The contents are desquamated epithelial keratin in concentric layers and cholesterol crystals in a solid-state [10]. MRI is the investigation of choice for epidermoids. These cysts are hypointense in T1-weighted images and markedly hyperintense in T2-weighted images. Diffusion-weighted Imaging (DWI) plays an essential role in distinguishing the cyst from other lesions [11].

The treatment of the choice for the tumor is surgical resection. The surgical approach depends on the extent of tumor involvement. The most commonly used approach is rectosigmoid (80%), followed by the combined middle cranial fossa and rectosigmoid approach (20%) [12]. In the present case, near-total resection was done as complete removal was a technical challenge due to the severely adherent tumor capsule to the CP angle dura. The patient was followed up for a mean duration of two years [11]. The prognosis of these cysts is excellent, but the recurrence rate is 8.3-25% [13]. The radiological confirmation of the same can be challenging as, after removal of the tumor, the remaining defect can fill with CSF that can resemble a signal of a recurrent lesion on CT scan or standard T2-weighted sequences [14].

## Conclusions

We report a rare case of a giant epidermoid cyst of the posterior fossa of the brain extending into the jugular foramen, left CP angle reaching up to the floor of the middle ear which was removed via suboccipital craniotomy extending up to the rectosigmoid junction. There was no preoperative complication except the weakness in the arms. This tumor is exclusively extradural, during surgery no breach in dura was observed. Near-total excision was performed and specifically at CP angle, the dura was badly thinned and the capsule adhered tightly which made the separation impossible without dural breach. There were no postoperative complications. Intracranial epidermoids are slow-growing benign cystic lesions, whose symptoms for compression or irritation of vascular or neurological structures become apparent later on. Surgical resection is the treatment of the choice for the tumor and it depends on the extent of the tumor involvement. It's important to follow-up since recurrences are commonly reported in such cases. 
